# Strategic Processing of Gender Stereotypes in Sentence Comprehension: An ERP Study

**DOI:** 10.3390/brainsci13040560

**Published:** 2023-03-27

**Authors:** Yanan Du, Yaxu Zhang

**Affiliations:** 1 School of Psychological and Cognitive Sciences and Beijing Key Laboratory of Behavior and Mental Health, Peking University, Beijing 100871, China; dyanan@pku.edu.cn; 2 Key Laboratory of Machine Perception (Ministry of Education), Peking University, Beijing 100871, China

**Keywords:** gender stereotypes, strategic processing, pragmatics, sentence comprehension, event-related brain potentials

## Abstract

Gender stereotypes are often involved in language comprehension. This study investigated whether and to what extent their processing is under strategic control, by examining both proportion and order effects related to gender stereotypes for role nouns. We manipulated stereotypical gender consistencies, as in “Li’s daughter/son was a nurse…”, the relative proportions of gender-consistent and gender-inconsistent sentences (80%:20% and 50%:50% for high-proportion and equal-proportion sessions, respectively), and a between-participant factor of session order (high-proportion sessions preceding equal-proportion sessions and a reversed order for the high–equal and equal–high groups, respectively). Linear mixed-effect models revealed a larger N400 and a larger late negativity for stereotypically inconsistent compared to consistent sentences for the high–equal group only. These results indicate that even if sentence contexts have already determined the gender of target role characters, gender stereotypes for role nouns are still activated when the first half of the experiment facilitates their activation. The analyses of trial-by-trial dynamics showed that the N400 effects gradually decreased throughout equal-proportion sessions for the equal–high group. Our findings suggest that the processing of gender stereotypes can be under strategic control. In addition, readers may develop other strategies based on sentence contexts, when the processing strategy based on cue validity is not available.

## 1. Introduction

Comprehenders often use their knowledge or beliefs about the properties of social groups during language comprehension. For example, when readers encounter a stereotypically female- or male-biased occupation name (e.g., “nurse” or “electrician”) in a sentence, they could activate the stereotypical gender of the target occupation character (via inferential processes based on world knowledge, according to Carreiras et al. [1]; see [2,3] for related evidence). If the accessed stereotypical gender of the occupation name is inconsistent with the definitional gender of another word that has a co-referential relationship with the occupation name, as in “The electrician taught herself…”, processing difficulties or gender inconsistency effects could occur. These difficulties can manifest themselves as longer self-paced reading times on target clauses [4]; longer eye-movement reading times such as first pass, second pass, and/or go-past times on target words (e.g., [2,5]); or a larger event-related potential (ERP) response such as an N400 (e.g., [6,7,8]) and/or a P600 (e.g., [8,9]) (though these ERP responses are not specific to stereotypical gender inconsistencies; for example, see [10] for the P600 response to grammatical gender violations). However, whether and to what extent gender stereotypes for role names are processed automatically or strategically during language comprehension remain unresolved issues. The present study aimed at investigating these issues.

So far, there has been evidence for an automatic or rapid activation of gender stereotypes during both lexical (e.g., [11,12,13,14]) and sentence or discourse processing (e.g., [4,6,7,8,15,16]). For example, participants were still slower in judging whether or not a role noun (e.g., “engineer”) and a kinship term (e.g., “mother”) could refer to the same person in an incongruent compared to a congruent condition, even if they were given a considerable amount of time (1,800 msec) or an explicit instruction to suppress gender stereotypes [12]. In addition, by using a masked priming technique, Pesciarelli et al. [13] provided both behavioral and neuroscientific evidence for an unconscious activation of gender stereotypes. They asked participants to judge the grammatical gender of an Italian third-person singular pronoun (“lui” [“he”] or “lei” [“she”]) and to ignore the preceding role noun (e.g., “insegnante” [“teacher”]). They found a longer reaction time and a larger N400 response to the pronouns when the preceding role nouns were gender-incongruent than when they were gender-congruent, even when the role nouns were presented only for 50 msec and were then immediately followed by a backward mask. The masked priming effects have been treated as strong and direct evidence for an unconscious activation of gender stereotypes during lexical processing.

Besides the word-pair studies discussed above, sentence processing studies employing an implicit task have also indicated the automaticity of gender stereotype processing (e.g., [7,8]). For example, Proverbio et al. [7,8] found that even if participants were asked to simply judge whether the sentence-final words represented an animal or not, there was still an early effect of gender incongruency: the sentence-final words elicited a larger N400 when they violated gender stereotypes, as in the Italian version of “Prepared the tomato sauce and then shaved”, compared to their congruent counterparts.

Interestingly, a few studies have provided evidence that could be used to address the degree of automaticity of gender stereotype processing or whether and the extent to which this type of processing is under comprehenders’ strategic control [2,3,11,12]. First, in Banaji and Hardin’s [11] word-pair priming study, in which role nouns (e.g., “secretary”) and pronouns (e.g., “he”) were used as prime and target words, respectively, stereotypical gender exhibited gender-priming effects only when the participants made gender decisions to pronouns; no priming was found when the participants performed a lexical decision task. This finding suggests that stereotypical gender information is not always accessed automatically, at least in the context of lexical processing (also see [2] for comments). Second, increasing the time available for processing occupation names and the instruction alerting the participants to the possible influence of gender stereotypes on word-pair judgment attenuated, though did not entirely eliminate, gender incongruity effects [12]. These results suggest that while the activation of gender stereotype is a very automatic process, it may be possible to strategically control it with voluntary effort in the context of lexical processing.

Most relevantly to the present work, Kreiner et al.’s [2] sentence processing study found that when a cataphoric reflexive preceded a role name and, thus, had already assigned a categorical gender to the role-named character, as in the cataphora sentence “After reminding herself about the letter, the minister immediately went to the meeting at the office”, the typical gender incongruity effects disappeared. They interpreted this finding as evidence that stereotypical inferences are no longer necessary and, thus, are prevented in cataphora sentences, resulting in the stereotypical gender information not being accessed and the effects of gender incongruity being eliminated. For this preventing account, the access of stereotypical gender information can be fully under readers’ strategic control, as long as the gender of the role name is determined in time (see [17] for a scenario in which the gender of the role name could not be determined in time to allow the preventing of stereotypical inferences, as in “After buying a new, comfortable strapless bra in the clothes store the politician/dressmaker went to meet a friend”; for comments, see [2]).

However, Kreiner et al.’s [3] ERP results seem to be inconsistent with the preventing or strategic control account that is discussed above. Kreiner et al. [3] argued for the existence of an early (250–400 ms) ERP effect for gender incongruity for the stereotypical role nouns (e.g., “minister”) in cataphora sentences. It thus seems that stereotypical gender inferences may not be prevented by constraining cataphoric reflexives (e.g., “herself”). Kreiner et al. [3] interpreted the early ERP effects for gender incongruity as reflecting an automatic activation of stereotypical genders followed by fast “gender-coercing” for role nouns, that is, forcing the automatically activated stereotypical gender to shift toward the gender of the preceding cataphoric reflexive. This interpretation is plausible, given that no such early effects occurred for definitional role nouns (e.g., “king”), for which coercion cannot occur (gender incongruencies for definitional role nouns elicited only a 650–800 ms ERP response, which could reflect gender mismatch processing in such nouns).

Although Kreiner et al.’s [3] coercing account has somewhat reduced the degree of strategic control that is believed to be involved in the processing of gender stereotypes, a closer look at their ERP results reveals that the early (250–400 ms) ERP effect was, in fact, not statistically reliable. First, the analyses of gender consistency by noun-type interaction performed at each region of interest (ROI) were not licensed by a statistically reliable three-way interaction of gender consistency, noun type, and ROI. Second, no simple effect tests were conducted; instead, only inspections of the mean amplitudes were performed. Therefore, Kreiner et al.’s ERP data actually failed to provide reliable evidence for their coercing account.

In Kreiner et al.’s [2,3] studies, the processes of strategic control (either completely preventing stereotypical gender inferences or gender coercing) underlying the sentence-level gender stereotype processing were indirectly inferred based on the influence of the preceding cataphoric reflexives on the activation of the stereotypical gender of subsequent role nouns. In contrast, the present study aimed to more directly investigate whether and to what extent the access of stereotypical gender for role names can be under strategic control during sentence comprehension.

The proportion paradigm has been shown to be a particularly useful tool for directly investigating strategic control processes during language comprehension (e.g., [18,19,20,21,22,23,24,25,26,27]). For example, in a self-paced reading experiment, Brothers et al. [18] manipulated not only the predictability of the critical word (CW) (predictable vs. unpredictable), such as “spider” in (1a) and (1b), but also the global validity of lexical prediction, that is, the proportion of sentences in which the CWs were predictable (87.5%, 50%, or 12.5%). The predictability effect manifested as shorter reading times in the critical region (the CW plus the adjacent spillover word), such as “spider on” in (1a) and (1b), when the CW was highly predictable, as in (1a), compared to when the CW was unpredictable, as in (1b). Crucially, the size of predictability effects decreased monotonically as the global validity of lexical prediction decreased. This finding suggests that readers dynamically modulate top–down anticipatory processing strategies based on the global validity of lexical prediction.

(1a) The web had been spun by the large spider on the porch.

(1b) Alex said he wanted to watch the large spider on the porch.

In the present study, we investigated whether and to what extent the use of social knowledge, such as gender stereotypes, is sensitive to top–down strategies during sentence comprehension, an issue that has not been well addressed (see [22] for an influence of strategic control on the use of world knowledge related to typical or atypical locations for a given object or event). We therefore used a consistency proportion paradigm. In this paradigm, we first manipulated the consistency between the definitional gender of head nouns (a kinship term) of the subject noun phrases and the stereotypical gender of the predicate nouns (a role name, the CWs for ERPs), as in the Chinese version of “Li’s daughter/son is a nurse and often works night shifts”. More crucially, we also manipulated the overall relative proportion of gender-consistent and gender-inconsistent sentences in a session by manipulating non-critical, filler sentences. Thus, the overall relative proportion was either 80%:20% for the session of the high proportion of consistent sentences (henceforth: high-proportion session) or 50%:50% for the session of the equal proportion of consistent and inconsistent sentences (henceforth: equal-proportion session). Both the consistency and the proportion were treated as within-subject factors. In addition, the order of two different sessions was manipulated as a between-subject factor (a high-proportion session followed by an equal-proportion session or a reversed order, i.e., the high–equal and the equal–high groups, respectively) to examine any possible dynamic modulation of the strategic control across the course of the experiment based on the change in the relative proportion of consistent and inconsistent sentences.

We expected both a larger N400 and a larger late negativity (LN) at the CWs (the role nouns) for the inconsistent compared to the consistent sentences, as was the case in some previous studies that also used stereotypical role nouns as CWs (e.g., [6,28]). More crucially, if the processing of gender stereotypes can indeed be under strategic control, then the gender inconsistency effects would be more likely to be observed when participants are encouraged to use the stereotypical gender cues, such as when the high-proportion session precedes the equal-proportion session; in this case, participants could gradually expect consistent instead of inconsistent sentences during the high-proportion session, and such a preconceived expectation could even influence the processing of gender stereotypes during the subsequent equal-proportion session.

Alternatively, if the processing of gender stereotypes is a highly automatic process, neither the proportion nor the order factors would modulate the gender inconsistency effects. In the present study, we test both possibilities above.

## 2. Methods

### 2.1. Participants

Fifty-four native Mandarin Chinese speakers gave informed consent to participate in the ERP experiment and were paid for their participation. All participants were right-handed, had normal or corrected-to-normal vision, and had no history of neurological or psychiatric disorder. In total, 6 participants were excluded from the analyses due to less than 30 (30/40 or 75%, see Section 2.2) artifact-free trials for one or more conditions, leaving 48 participants (24 females; mean ± S.D. age = 22.38 ± 2.89 years; age range = 18–29 years) for the reported analyses. The 24 male and 24 female participants were randomly assigned to the high–equal (12 females; mean ± S.D. age = 22.38 ± 2.78 years; age range = 19–29 years) and the equal–high groups (12 females; mean ± S.D. age = 22.38 ± 3.12 years; age range = 18–30 years). This study was approved by the Human Subject Review Committee at Peking University.

### 2.2. Materials and Normative Measures

The critical materials consisted of 160 sets of Chinese 2-clause sentences, with the first clause being the target clause in which the definitional gender of the head noun (a kinship term) of the subject noun phrase was either consistent or inconsistent with the stereotypical gender of the predicate noun (a role name, the CW for ERPs) (see Table 1 for examples). Both the kinship terms (20 female and 20 male) and role nouns (20 stereotypically female and 20 stereotypically male) were the same as those used by Du and Zhang [28] (see [28] for the other detailed properties of these items). The second clause was added to avoid the CWs occurring in sentence-final positions where sentence-final wrap-up processes (a summary or global processing of the whole sentence) could influence the target ERPs (see [29] for a direct comparison of the ERPs in sentence-internal and sentence-final positions). Within each set of critical items, the consistent and inconsistent sentences differed only in the kinship term (e.g., “daughter” and “son” in Table 1).

The 160 sets of critical sentences were assigned to two experimental lists by using a Latin square procedure. Then, each list was evenly split into two sessions (i.e., the high-proportion and equal-proportion sessions), with each containing the same set of role nouns. We manipulated the overall relative proportion of consistent and inconsistent sentences in a specific session (80%:20% and 50%:50% for the high-proportion and the equal-proportion sessions, respectively) by manipulating filler sentences, which had the same sentence construction as the critical items. As shown in Table 1, for the high-proportion session, a set of 80 critical items (40 for each level of consistency) was combined with a separate set of 120 gender-consistent fillers, while for the equal-proportion session, the other set of 80 critical items (40 for each level of consistency) were combined with another separate set of 120 fillers (60 consistent and 60 inconsistent). These fillers above were created by using 40 stereotypically male or female role names (20 for each type). A pretest conducted by Du and Zhang [28] showed that as an index reflecting the degree of gender stereotypes, the mean (SD) percentage of males or females in the population who engage in a specific role was 83.88% (12.76%) and 74.13% (16.63%) for these stereotypically male and female role names, respectively. Note that these role names had a similar level of gender stereotypes to those used in the critical items (see [28]).

In addition, to offset the highly similar syntactic structure and the extremely limited types of anomalies (gender inconsistencies) both in the critical items and in the fillers described above, another separate set of 200 filler sentences of various constructions was added to both the high-proportion and the equal-proportion sessions. These fillers also contained two clauses but did not involve gender stereotypes. Half of them contained various syntactic and semantic anomalies. Therefore, for each session in each list, the 80 critical items were pseudo-randomly mixed with 320 fillers. Finally, we manipulated the order of the two sessions (either the high-proportion session prior to the equal-proportion session or the reverse), resulting in four experimental lists totally.

We performed an online pretest of the plausibility of the target clauses by using “wjx” powered by “www.wjx.cn” (accessed on 15 July 2020) to validate our manipulation of gender consistency. The items included in this pretest were the same as those used in the ERP experiment, except that only the target/initial clauses (e.g., the Chinese version of “Li’s son is a nurse, …”) instead of the whole sentences were presented. A separate group of 34 native Chinese speakers (17 females; mean ± S.D. age = 22.68 ± 2.57 years; age range = 18–28 years) rated each critical clause for plausibility on a 5-point Likert scale, in which “1” means “completely implausible” and “5” means “completely plausible”. Data from two participants were excluded from the analyses, due to their extremely quick (less than 15 min) or slow (about 2.5 h) performance.

The ratings of the remaining 32 participants (half females; mean ± S.D. age = 22.63 ± 2.57 years; age range = 18–28 years) were analyzed with cumulative link mixed models (CLMMs) with the clmm2 function in the ordinal package (version 2019.12-10) [30] in R (version 4.0.2) [31]. All three categorical factors (consistency, proportion, and order) were coded by using sum coding [32]. The models started with maximal random-effect structures [33] and were simplified by removing the random effects stepwise until convergence. The random effects of the best model included by-subject random intercepts and random slopes for consistency and proportion and by-item random intercepts and random slopes for consistency and order. The CLMMs revealed a significant main effect of consistency (β = 1.12, *SE* = 0.18, *z* = 6.34, *p* < 0.001), with the inconsistent target clauses (*M* = 4.34, *SD* = 0.99) being rated less plausible than the consistent ones (*M* = 4.72, *SD* = 0.57), as expected. There were no significant interactions involving the factor consistency (*p*s ≥ 0.085; for more details, see Table S1).

Finally, to address the potential influence of the cloze probabilities for the CWs on the target ERPs, we performed an online sentence completion test by using “wjx” powered by “www.wjx.cn” (accessed on 15 July 2020). In this test, another separate group of 32 native Chinese-speaking students (16 females; mean ± S.D. age = 20.66 ± 1.71 years; age range = 18–27 years) was presented with the clause fragments up to but not including the CWs and was asked to continue each clause fragment with the first word that came to mind. The 160 sets of critical clause fragments were assigned to two test lists by using a Latin square procedure. To offset the highly similar sentence structures in the critical items, for each list, the 160 critical items were pseudo-randomly mixed with 160 filler sentence fragments of various constructions. Each participant received only one list. The mean (SD) cloze probabilities of the CWs (or their synonyms) were 2.42% (15.38%) and 0.82% (9.02%) for the consistent and inconsistent conditions, respectively. The cloze probability was used as a predictor of the ERP amplitudes to statistically control for the potential influences of the cloze probabilities on the ERPs of interest (see Section 3).

### 2.3. Procedure

The participants sat on a chair in front of the screen at a distance of approximately 100 cm in a quiet, dimly lit room. The experimental stimuli were displayed in white characters on a black background and in the center of the screen. Each trial started with an 800 ms fixation, followed by a 500 ms blank interval. After that, the sentences were presented segment-by-segment (a word or a short phrase) at a rate of 800 ms per segment (400 ms segment and 400 ms blank interval, except for the final segment, which was followed by an 800 ms blank). The participants were advised to try to avoid blinking during the presentation of the sentence stimuli in order to reduce eye movements during the critical epochs. Then, a yes/no comprehension question appeared in one-fourth of the time. Participants were asked to read the sentences carefully and answer the questions by pressing the “D” or “K” keys on a keyboard. The response keys were counterbalanced across the participants. The questions remained on the screen until the participant gave a response but for a maximum of 3 s. The next trial began after a 2 s interval.

Each participant received only one of the four lists. For each list, the total 800 sentences were evenly divided into ten blocks, with a 3–5 min break between them. The participants completed 40 practice trials before the formal experiment. The experiment lasted about 2.5 h.

### 2.4. EEG Recording and ERP Data Analysis

The electroencephalogram (EEG) was recorded from 64 Ag/AgCI electrodes mounted in an elastic cap (EASYCAY GmbH, Worthsee-Etterschlag, Germany). The EOG activities were recorded from electrodes placed at the outer canthus of each eye, and above and beneath the right eye. Electrode impedances were kept below 5 kΩ. EEG signals were amplified by using BrainAmps amplifiers (Brain Products, Munich, Germany) with an online bandpass of 0.016–100 Hz at a sample rate of 1 kHz. The EEG recordings were referenced online to the electrode attached to the nose.

Raw EEG data were preprocessed with the BrainVision Analyzer Version 2.0 (Brain Products, Munich, Germany). Continuous EEG data were down-sampled to 250 Hz, were re-referenced to the average of the mastoids (TP9 and TP10), and were bandpass-filtered between 0.1 and 40 Hz by using a zero-phase IIR Butterworth filter (24 dB/oct). The ocular artifacts were corrected by using independent component analysis (ICA) methods. The epoch length was 1200 ms, ranging from 200 ms before to 1000 ms after the onset of the CW (role noun). The epochs with amplitudes above ±75 μV were excluded from the analyses, resulting in an average of 8.03% (range: 4.48% to 11.05%) trials per condition being excluded.

Note that as shown in Table 1, the sentence contexts prior to the CWs differed in the kinship terms between the consistent and the inconsistent conditions, which could result in both spillover effects and baseline artifacts in the target ERPs if traditional baseline correction is performed (see [34,35]). Therefore, we did not perform traditional baseline correction during the data preprocessing. Instead, to statistically control for potential contextual confounds, single-trial ERP amplitudes, which were extracted by using EEGLAB (v.2019.0) [36], were analyzed with linear mixed-effects models, by using the lmer function in the packages lme4 (v.1.1–26) [37] and lmerTest (v.3.1–2) [38] for R (v. 4.0.2) [31]. Crucially, the scaled trial-wise mean amplitude in the 200 ms pre-CW interval was included as a predictor for target ERPs, as suggested by Alday [39]. In addition, the categorical variables were coded by using sum coding [32], and the continuous variables were z-transformed.

All models began with a maximal random effects structure [33] and were simplified by removing random effects based on the likelihood ratio test results until the best model fit was obtained [40] (see Section 3 for the fixed effects included in the models for different analyses). We performed Type II Wald chi-square tests by using the ANOVA function in the car R package (v.3.0–9) [41] to obtain p values. When applicable, post hoc pairwise comparisons of estimated marginal means were conducted by using the emmeans R package (v.1.5.1) [42]. Bonferroni correction was applied, and corrected p-values are reported for multiple tests.

## 3. Results

The mean ± SD accuracy of answering yes/no comprehension questions was 94.64% ± 3.00%, suggesting that the participants read the sentences attentively.

Based on the visual inspection, we chose 2 time windows for the statistical analysis: (a) 300–600 ms for N400 effects and (b) 650–1000 ms for LN effects. In addition, we selected a group of eight central channels (C1, Cz, C2, CP3, CP1, CPz, CP2, and CP4) for N400 effects, and another group of eight central channels (FC1, FC2, C1, Cz, C2, CP1, CPz, and CP2) for LN effects, based on both visual inspections and the consideration of symmetries.

**Figure 1:**
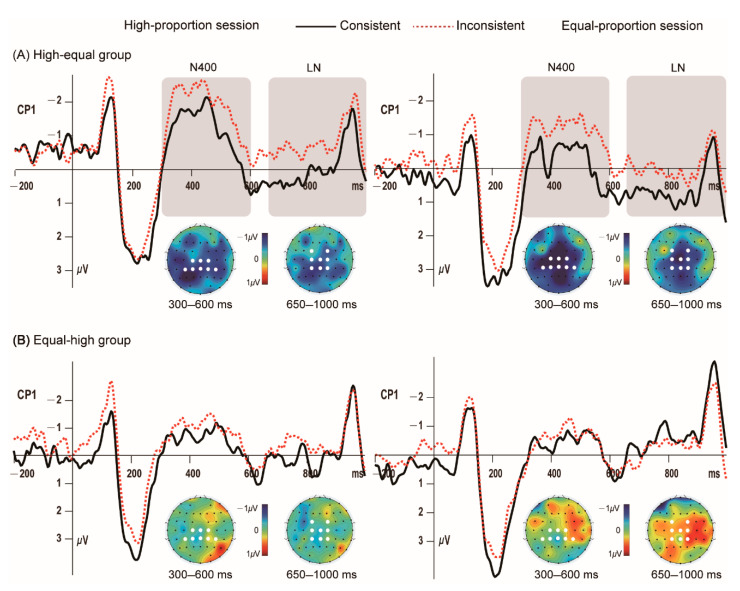
ERPs time-locked to the onset of the role nouns. Grand-average ERP waveforms at CP1, a representative channel, for the consistent and the inconsistent conditions in both high-proportion and equal-proportion sessions for the high–equal (**A**) and the equal–high groups (**B**), respectively. The scalp topographies of the corresponding difference waves (inconsistent minus consistent) are shown for both N400 and late negativity (LN) time windows. The white dots within each topography indicate the locations of all eight channels chosen for data analyses.

Figure 1 illustrates both the grand average ERPs elicited by the role nouns at CP1, a representative channel, for all eight conditions, and the scalp topographies of the difference waves of the inconsistent minus the consistent conditions in the two time windows. As shown in Figure 1, for the high–equal group, the role nouns elicited both a larger N400 in the 300–600 ms range and a larger LN in the 650–1000 ms range in the inconsistent compared to the consistent conditions for both the high-proportion and the equal-proportion sessions. Interestingly, there were no obvious ERP effects for gender consistency in both sessions for the equal–high group. The analyses using mixed-effect models statistically confirmed the visual impressions above.

### 3.1. The 300–600 ms Time Window

For this early time window, the fixed effects included all three factors that we manipulated (consistency, proportion, and order), the mean amplitude in the 200 ms pre-CW baseline interval, cloze probability, and all possible interactions. The random effects included by-subject and by-item random intercepts and by-subject random slopes for proportion. Table 2 shows the estimated marginal means and standard errors of both N400 and LN amplitudes for all eight conditions. As shown in Table 3 (see Tables S2 and S3 for full model summaries), there was an interaction of consistency with order. Post hoc pairwise comparisons of estimated marginal means showed that the role nouns elicited a larger N400 in the inconsistent condition (*M* = −1.26 μV, *SE* = 0.39, 95% CI [−2.03, −0.49]) compared to the consistent condition (*M* = −0.55 μV, *SE* = 0.38, 95% CI [−1.31, 0.22]) for the high–equal group, β = −0.08, *SE* = 0.03, 95% CI [−0.14, −0.03], *t* = −3.10, *p* = 0.004, but not for the equal–high group, β = 0.02, *SE* = 0.03, 95% CI [−0.03, 0.07], *t* = 0.70, *p* = 1.

In addition, although there was a three-way interaction of consistency, order, and cloze probability, further analyses showed that the amplitude of the N400 did not vary with the cloze probability of the CW for all four conditions (*p*s > 0.158), suggesting that the predictabilities of the role nouns did not influence the magnitudes of the N400 effects in our experiment. This was not unexpected, given that the mean cloze probabilities of the CWs (or their synonyms) were very low (only 2.43% and 0.82% for the consistent and inconsistent conditions, respectively; see Section 2.2).

**Figure 2:**
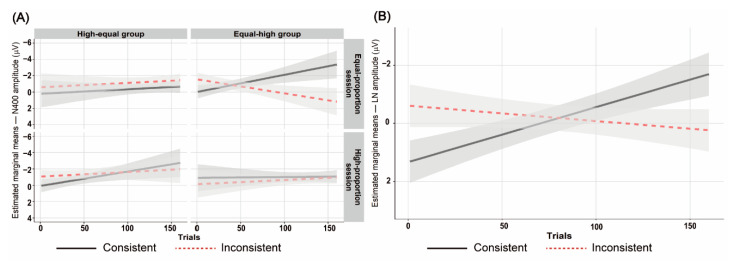
Estimated marginal means for N400 (**A**) and LN (**B**) amplitudes for role nouns. (**A**) The model-based estimates of N400 amplitudes by trials, consistency, session, and order. (**B**) The model-based estimates of LN amplitudes by trials and consistency. Ribbons represent 83% confidence intervals, the non-overlap of which corresponds to a 5% significance level (see [46]).

To further look at the neural dynamics of the N400 effects, we analyzed the trial-by-trial dynamics for this early time window. We thus additionally included fixed effects involving trials, which contained both the main effects of the trials and the interactions of the trials with other factors, as in some previous studies (e.g., [23,26,43,44,45]). We did not include the interaction between trials and cloze probability for model parsimony. The random effects in the best model included by-subject and by-item intercepts and by-subject random slopes for proportion. As shown in Table 4 (see Tables S4 and S5 for full model summaries), there was a 4-way interaction among consistency, proportion, order, and trials. Further trend analyses showed that the N400 effect decreased over the equal-proportion session for the equal–high group (see Figure 2A), β = 0.01, *SE* = 0.00, 95% CI [0.00, 0.01], t = 2.85, *p* = 0.018. No other significant trends were found (*p*s = 1).

### 3.2. The 650–1000 ms Time Window

For this late time window, we analyzed the same fixed effects as those analyzed for the early (300–600 ms) time window. The random effects for the best model included by-subject and by-item random intercepts and by-subject random slopes for proportion. As shown in Table 5 (for full model summaries, see Tables S6 and S7), there was an interaction of consistency with order. Post hoc pairwise comparisons of estimated marginal means revealed a larger LN at the role nouns for the inconsistent (*M* = −0.20 μV, *SE* = 0.35, 95% CI [−0.90, 0.50]) compared to the consistent conditions (*M* = 0.54 μV, *SE* = 0.34, 95% CI [−0.15, 1.22]) for the high–equal group, β = −0.09, *SE* = 0.03, 95% CI [−0.15, −0.03], *t* = −2.83, *p* = 0.009, but not for the equal–high group, β = 0.02, *SE* = 0.03, 95% CI [−0.04, 0.07], *t* = 0.49, *p* = 1. These results echoed the findings in the N400 time window. Thus, the factor order could have a similar influence on the early and later ERP responses.

To look at the more detailed neural dynamics of the LN effects, we analyzed the trial-by-trial dynamics for this time window, as we did for the early (300–600 ms) time window. For the best model, the random effects contained by-subject and by-item random intercepts and by-subject random slopes for proportion. As shown in Table 6 (for full model summaries, see Tables S8 and S9), there was an interaction between consistency and trials. A further trend analysis revealed that the LN effect decreased throughout the experiment (see Figure 2B), β = 0.003, *SE* = 0.001, 95% CI [0.001, 0.005], *t* = 3.20, *p* = 0.001. In addition, although there was a significant three-way interaction among consistency, proportion, and order, post hoc pairwise comparisons of estimated marginal means did not reveal any statistically reliable effects of consistency.

## 4. Discussion

The current study aimed to more directly explore whether and to what extent the processing of gender stereotypes for role names are under strategic control during Chinese sentence reading. We used a consistency proportion paradigm, in which we manipulated three factors, that is, consistency, proportion, and order. In the early (300–600 ms) time window, the role nouns evoked a larger N400 in the inconsistent than in the consistent conditions for both the high-proportion and the equal-proportion sessions in the high–equal group. The N400 effects for gender-inconsistent stereotypical role nouns, which were also observed in the previous literature (e.g., [6,28]), highly likely reflect an early detection of gender inconsistencies and/or difficulty in retrieving or integrating lexical information. In contrast, there were no N400 effects for gender inconsistencies in any session for the equal–high group. It thus appears that the order of different proportion sessions did determine the presence or absence of the N400 effects.

The scenario of N400 effects above is expected if gender stereotype processing can be under strategic control. Recall that for the high-proportion session, the overall relative proportion of gender-consistent and gender-inconsistent sentences was 80%:20%, which could encourage participants to access and use the stereotypical gender cues, such that a specific processing strategy could be developed. When the high-proportion session preceded the equal-proportion session, which is the case for the high–equal group, participants could continue to employ the strategy that they had developed during the preceding session while reading sentences in the equal-proportion session, even if the stereotypical gender cues were valid only 50% of the time.

The absence of the N400 effects for any session as a whole for the equal–high group appears to be congruent with Kriener et al.’s [2] preventing account, which we have discussed in Section 1. According to this account, the access of stereotypical gender information for a role noun can be completely prevented, as long as the gender of the role noun has been determined in the preceding sentence context, for example, by a cataphoric reflexive [2]. In our experiment, the kinship terms in the sentence contexts had already determined the gender of the role nouns, as in the Chinese version of “Li’s son is a nurse, …”, such that the access of the stereotypical gender was no longer necessary and, thus, was prevented. However, Kriener et al.’s [2] preventing account alone cannot explain the presence of the N400 effects for both sessions in the high–equal group.

Interestingly, whereas the high-proportion session encouraged the participants in the high–equal group to access and use the stereotypical gender cues, it did not encourage the participants in the equal–high group to access and use such cues. The latter scenario occurred probably because the high-proportion session was preceded by the equal-proportion session, in which Kriener et al.’s [2] preventing strategy had been developed, and the participants continued to use such a strategy even during the high-proportion session. Thus, although the overall relative proportion of gender-consistent and gender-inconsistent sentences does shape the strategies of using stereotypical gender cues in accordance with statistical rules during sentence processing, it plays a limited role when other processing strategies, such as the preventing strategy based on preceding sentence contexts, have already been employed earlier.

In addition, our finding that the N400 effect decreased over the equal-proportion session for the equal–high group also provides evidence that the participants in this group could gradually develop the preventing strategy on the basis of sentence context information, when there were no strategies based on the statistical rules available.

In the late (650–1000 ms) time window, we found an LN effect for gender inconsistencies in both sessions for the high–equal group. The LN effects have also been observed in some previous studies on the processing of stereotypical gender inconsistencies (e.g., [6,7,28]) or other types of pragmatic anomalies (e.g., [47,48,49]). In the present study, the LN effects may reflect a reinterpretation process that is necessary in the face of gender inconsistencies, probably by suppressing the prevalent gender stereotype and switching to an alternative interpretation for role names (e.g., “actually, a male can be a nurse…”), a process similar to the gender coercing proposed by Kriener et al. [3]. Given that the reinterpretation process may also underlie other types of language processing such as the resolution of syntactic and/or semantic ambiguities (e.g., [50]), future studies need to address whether the LN effects reflect a general process of reinterpretation.

More crucially, unlike the high–equal group, the equal–high group did not show an LN effect for gender inconsistencies in any session. These results were congruent with those obtained in the N400 time window, suggesting that the session order might have a similar influence on the early and later ERP responses to gender inconsistencies. Our finding that participants dynamically adjusted their processing of gender stereotypes based on the validity of stereotypical cues appears to be congruent with the good-enough approach to language processing (e.g., [51,52]). When there was a highly valid stereotypical cue available (such as in the high-proportion session for the high–equal group), the participants could use a good-enough representation, which was formed in accordance with the stereotypical gender cue, to interpret both the consistent and inconsistent sentences (see [50] for evidence that Russian speakers use a good-enough representation based on animacy cue to interpret syntactically ambiguous sentences). In this way, both N400 and LN effects occurred for the high–equal group but not for the equal–high group. In addition, the reinterpretation account for the LN effects could be also congruent with the good-enough approach, in which the participants could use a good-enough representation such as “actually, a male can be a nurse…” to reinterpret the inconsistent sentences such as “Li’s son is a nurse, …”.

Somewhat unexpectedly, the analyses of the trial-by-trial dynamics for the LN time window showed that the LN effects decreased over trials for any sessions in any groups. This pattern differed from that observed for the N400 effects, indicating that the processes reflected by the LN effects are not as sensitive to the factors of session and order as those reflected by the N400 effects. The exact mechanisms underlying such differences between N400 and LN effects are beyond the goal of the present study. We look forward to future studies investigating this issue.

The results of the present study, together with those of a previous study that examined how a strategic control affects the use of world knowledge related to typical or atypical locations for a given object or event [22], provide evidence that the use of world knowledge can be under strategic control during sentence processing. Note that although the present study has examined how processing strategies and sentence contexts interact and, thus, influence the access and use of social knowledge such as gender stereotypes for role nouns, the sentence context information was provided by a kinship term, a very particular word. In addition, we admit that our participants could have a specific social or cultural background. It is not clear whether the present findings can apply to both other types of sentence materials and other social or cultural backgrounds. We look forward to future research addressing these issues.

## 5. Conclusions

The present study aimed to investigate whether and to what extent the use of social knowledge, such as gender stereotypes, is under readers’ strategic control during sentence comprehension. Our results suggest that readers can develop a processing strategy based on the validity of the stereotypical gender cues, such that the access and use of social knowledge can be under their strategic control during sentence processing. In addition, readers may develop other strategies, for example, a strategy of preventing stereotypical inferences on the basis of sentence context information, when the processing strategy based on statistical rules (cue validity) is not available. Future studies need to further explore how different types of processing strategies interplay and, thus, influence the online use of social knowledge during sentence comprehension in different social or cultural backgrounds.

## Figures and Tables

**Table 1:** Examples for four types of sentences in the event-related potential (ERP) experiment, together with the corresponding number of trials for both high-proportion and equal-proportion sessions. Examples are given in Chinese, with English glosses and translations. The critical words are in bold.

	Consistency	Example Sentences	Number of Trials (for Each Order)	
			High-Proportion Sessions	Equal-Proportion Sessions
Critical	Consistent	老李的/女儿/是/一名/**护士**,/经常/值/夜班。	40	40
		Li’s/daughter/is/a/**nurse**,/often/works/night shifts. Li’s daughter is a **nurse** and often works night shifts.		
	Inconsistent	老李的/儿子/是/一名/**护士**,/经常/值/夜班。	40	40
		Li’s/son/is/a/**nurse**,/often/works/night shifts. Li’s son is a **nurse** and often works night shifts.		
Filler	Consistent	小张的/爸爸/是/一位/企业家，/在当地/颇有/声望。	120	60
		Zhang’s/father/is/an/entrepreneur,/locally/is well-known. Zhang’s father is an entrepreneur who is well-known locally.		
	Inconsistent	老宋的/堂妹/曾是/一名/采煤工，/年轻时/吃了/不少苦。	0	60
		Song’s/cousin [female]/was/a/coal miner,/when she was young/suffered a lot. Song’s cousin [female] was a coal miner and suffered a lot when she was young.		

**Table 2:** Estimated marginal means (*M*, μV) and standard errors (*SE*) of N400 and LN amplitudes as a function of order, proportion, and consistency.

			N400		LN	
Order	Proportion	Consistency	*M*	*SE*	*M*	*SE*
high–equal	high	consistent	−0.64	0.41	0.44	0.38
		inconsistent	−1.28	0.41	−0.20	0.37
	equal	consistent	−0.46	0.42	0.63	0.40
		inconsistent	−1.23	0.44	−0.20	0.43
equal–high	high	consistent	−1.02	0.41	−0.14	0.37
		inconsistent	−0.76	0.41	−0.10	0.37
	equal	consistent	−0.88	0.42	−0.80	0.40
		inconsistent	−0.84	0.44	−0.59	0.43

**Table 3:** Estimates of fixed effects and analysis of deviance (Type II Wald chi-square tests) for the N400 (300–600 ms) time window.

	β	*SE*	95% CI	*t*	χ^2^	*p* (>χ^2^)
intercept	0.00	0.03	[–0.06, 0.06]	−0.11		
consistency	0.02	0.01	[0.00, 0.04]	1.72	2.25	0.134
proportion	0.00	0.01	[–0.03, 0.02]	−0.37	0.55	0.457
order	0.00	0.03	[–0.06, 0.06]	−0.05	0.02	0.884
cloze	0.00	0.02	[–0.03, 0.03]	−0.17	0.67	0.413
consistency:proportion	−0.01	0.01	[–0.02, 0.01]	−0.54	0.05	0.816
consistency:order	0.03	0.01	[0.01, 0.04]	2.71	8.29	0.004
proportion:order	0.00	0.01	[–0.02, 0.02]	−0.24	0.12	0.729
consistency:cloze	0.01	0.02	[–0.02, 0.04]	0.71	0.004	0.947
proportion:cloze	0.02	0.02	[–0.01, 0.05]	1.10	0.21	0.645
order:cloze	0.00	0.02	[–0.03, 0.03]	0.08	0.84	0.358
consistency:proportion:order	0.00	0.01	[–0.02, 0.02]	0.14	0.07	0.793
consistency:proportion:cloze	−0.02	0.02	[–0.05, 0.01]	−1.08	0.82	0.365
consistency:order:cloze	−0.02	0.02	[–0.05, 0.01]	−1.09	3.92	0.048
proportion:order:cloze	0.00	0.02	[–0.03, 0.03]	−0.08	0.22	0.636
consistency:proportion:order:cloze	−0.01	0.02	[–0.03, 0.02]	−0.34	0.06	0.808

**Table 4:** Estimates of fixed effects and analysis of deviance (Type II Wald chi-square tests) for the N400 (300–600 ms) time window for models that included trials as a predictor.

	β	*SE*	95% CI	*t*	χ^2^	*p* (>χ^2^)
intercept	−0.01	0.03	[–0.08, 0.06]	−0.29		
consistency	−0.02	0.02	[–0.05, 0.02]	−0.82	2.17	0.141
proportion	−0.02	0.02	[–0.06, 0.02]	−0.96	1.31	0.252
order	−0.01	0.04	[–0.08, 0.06]	−0.29	0.18	0.673
cloze	0.00	0.02	[–0.03, 0.03]	−0.14	1.21	0.272
trials	−0.03	0.02	[–0.07, 0.01]	−1.50	2.23	0.135
consistency:proportion	0.01	0.02	[–0.03, 0.04]	0.39	0.52	0.472
consistency:order	0.04	0.02	[0.01, 0.08]	2.40	5.68	0.017
proportion:order	−0.03	0.02	[–0.07, 0.01]	−1.40	2.08	0.149
consistency:cloze	0.02	0.02	[–0.02, 0.05]	0.96	0.11	0.741
consistency:trials	−0.03	0.02	[–0.07, 0.00]	−1.70	2.63	0.105
proportion:cloze	0.02	0.02	[–0.01, 0.05]	1.20	0.24	0.623
proportion:trials	−0.01	0.02	[–0.05, 0.03]	−0.51	0.30	0.585
order:cloze	0.00	0.02	[–0.03, 0.03]	0.29	0.52	0.473
order:trials	−0.02	0.02	[–0.05, 0.02]	−0.90	0.81	0.369
consistency:proportion:order	−0.03	0.02	[–0.06, 0.01]	−1.40	1.68	0.194
consistency:proportion:cloze	−0.02	0.02	[–0.05, 0.01]	−1.20	1.16	0.282
consistency:proportion:trials	0.02	0.02	[–0.01, 0.06]	1.15	1.25	0.263
consistency:order:cloze	−0.02	0.02	[–0.05, 0.01]	−1.32	3.94	0.047
consistency:order:trials	0.02	0.02	[–0.02, 0.05]	0.81	0.96	0.327
proportion:order:cloze	0.00	0.02	[–0.03, 0.03]	−0.17	0.06	0.804
proportion:order:trials	−0.01	0.02	[–0.04, 0.03]	−0.38	0.16	0.686
consistency:proportion:order:cloze	0.00	0.02	[–0.03, 0.03]	0.00	0.004	0.953
consistency:proportion:order:trials	−0.04	0.02	[–0.07, 0.00]	−2.03	4.27	0.039

**Table 5:** Estimates of fixed effects and analysis of deviance (Type II Wald chi-square tests) for the LN (650–1000 ms) time window.

	β	*SE*	95% CI	*t*	χ^2^	*p* (>χ^2^)
intercept	−0.01	0.03	[–0.06, 0.05]	−0.20		
consistency	0.02	0.01	[0.00, 0.04]	1.68	1.34	0.247
proportion	0.01	0.01	[–0.01, 0.04]	1.23	0.41	0.525
order	0.03	0.03	[–0.02, 0.09]	1.27	0.93	0.336
cloze	−0.04	0.02	[–0.07, −0.01]	−2.36	1.85	0.173
consistency:proportion	0.00	0.01	[–0.02, 0.02]	−0.05	0.27	0.606
consistency:order	0.03	0.01	[0.00, 0.05]	2.36	6.18	0.013
proportion:order	−0.02	0.01	[–0.04, 0.00]	−1.71	2.92	0.087
consistency:cloze	0.02	0.02	[–0.01, 0.06]	1.38	0.04	0.840
proportion:cloze	0.04	0.02	[0.01, 0.07]	2.30	1.91	0.167
order:cloze	0.00	0.02	[–0.03, 0.03]	−0.04	1.63	0.201
consistency:proportion:order	−0.01	0.01	[–0.03, 0.02]	−0.48	0.23	0.633
consistency:proportion:cloze	−0.03	0.02	[–0.06, 0.01]	−1.61	1.19	0.276
consistency:order:cloze	−0.02	0.02	[–0.05, 0.02]	−0.95	1.43	0.232
proportion:order:cloze	−0.01	0.02	[–0.04, 0.02]	−0.61	0.45	0.504
consistency:proportion:order:cloze	0.01	0.02	[–0.03, 0.04]	0.37	0.51	0.473

**Table 6:** Estimates of fixed effects and analysis of deviance (Type II Wald chi-square tests) for the LN (650–1000 ms) time window for models that included trials as a predictor.

	β	*SE*	95% CI	*t*	χ^2^	*p* (>χ^2^)
intercept	−0.01	0.03	[–0.08, 0.05]	−0.42		
consistency	0.00	0.02	[–0.04, 0.04]	−0.02	1.27	0.259
proportion	0.02	0.02	[–0.02, 0.06]	1.03	0.56	0.455
order	0.02	0.03	[–0.04, 0.08]	0.63	0.17	0.679
cloze	−0.04	0.02	[–0.07, 0.00]	−2.21	0.59	0.442
trials	−0.04	0.02	[–0.08, 0.00]	−1.80	3.09	0.079
consistency:proportion	0.01	0.02	[–0.03, 0.05]	0.32	0.62	0.431
consistency:order	0.02	0.02	[–0.02, 0.06]	1.07	1.19	0.275
proportion:order	−0.05	0.02	[–0.09, −0.01]	−2.43	5.68	0.017
consistency:cloze	0.03	0.02	[–0.01, 0.06]	1.67	0.36	0.551
consistency:trials	−0.07	0.02	[–0.11, −0.03]	−3.20	9.57	0.002
proportion:cloze	0.04	0.02	[0.01, 0.08]	2.46	2.52	0.112
proportion:trials	−0.02	0.02	[–0.06, 0.03]	−0.75	0.60	0.439
order:cloze	0.00	0.02	[–0.03, 0.03]	0.00	1.51	0.220
order:trials	0.01	0.02	[–0.03, 0.05]	0.45	0.18	0.675
consistency:proportion:order	−0.06	0.02	[–0.10, −0.02]	−2.95	8.72	0.003
consistency:proportion:cloze	−0.03	0.02	[–0.06, 0.01]	−1.64	1.33	0.248
consistency:proportion:trials	0.00	0.02	[–0.04, 0.04]	−0.20	0.05	0.827
consistency:order:cloze	−0.02	0.02	[–0.05, 0.02]	−1.05	1.51	0.220
consistency:order:trials	0.01	0.02	[–0.03, 0.05]	0.43	0.27	0.603
proportion:order:cloze	−0.01	0.02	[–0.04, 0.02]	−0.59	0.28	0.595
proportion:order:trials	−0.01	0.02	[–0.05, 0.03]	−0.43	0.20	0.656
consistency:proportion:order:cloze	0.01	0.02	[–0.03, 0.04]	0.49	0.67	0.414
consistency:proportion:order:trials	−0.02	0.02	[0.06, 0.02]	−1.09	1.22	0.269

## Data Availability

The data presented in this study are available on request from the corresponding author.

## Supplementary Materials

The following supporting information can be downloaded at: https://www.mdpi.com/article/10.3390/brainsci13040560/s1, Table S1: Summary of cumulative link mixed model fitted with the Laplace approximation for the rating scores in the pretest of plausibility. Table S2: Summary of linear mixed effect model for the N400 (300–600 ms) time window. Table S3: Analysis of deviance (Type II Wald chi-square tests) for the N400 (300–600 ms) time window. Table S4: Summary of linear mixed effect model with trials as an additional predictor for the N400 (300–600 ms) time window. Table S5: Analysis of deviance (Type II Wald chi-square tests) in the N400 (300–600 ms) time window for models with trials as an additional predictor. Table S6: Summary of linear mixed effect model for the LN (650–1000 ms) time window. Table S7: Analysis of deviance (Type II Wald chi-square tests) for the LN (650–1000 ms) time window. Table S8: Summary of linear mixed effect model with trials as an additional predictor for the LN (650–1000 ms) time window. Table S9: Analysis of deviance (Type II Wald chi-square tests) in the LN (600–1000 ms) time window for models with trials as an additional predictor.
