# Supplementary material for: Strategic Processing of Gender Stereotypes in Sentence Comprehension: An ERP Study
Source: Brain Sci. 2023 Mar 27;13(4):560. doi: 10.3390/brainsci13040560 (PMC10136608; doi:10.3390/brainsci13040560)
Supplement: Supplementary file 1 [file brainsci-13-00560-s001.zip › brainsci-2192921-supplementary.pdf]

Supplementary materials

Table S1: Summary of cumulative link mixed model fitted with the Laplace approximation for the rating scores in the pretest of plausibility. Summary of model produced by the call `clmm (formula = rating ~ Consistency * Proportion * Order + (Consistency + Proportion | subject) + (Consistency + Order | item), data = cv_rating_clm`

link	threshold	nobs	logLik	AIC	niter	max.grad	cond.H
logit	flexible	5120	-2794.92	5635.85	4354(52424)	1.30e-03	6.5e+02

Random effects:

Groups	Name	Variance	Std.Dev.	Corr	
item	(Intercept)	0.37097	0.6091		
	Consistency[consistent]	0.36790	0.6065	-0.972	
	Order[High-equal]	0.05098	0.2258	-0.219	-0.015
subject	(Intercept)	8.20372	2.8642		
	Consistency[consistent]	0.51182	0.7154	0.128	
	Proportion[High]	0.08532	0.2921	0.129	0.449

Number of groups: item 160, subject 32

Coefficients:

	Estimate	Std. Error	z value	Pr(>|z|)
Consistency[consistent]	1.11873	0.17652	6.338	2.33e-10
Proportion[High]	0.04240	0.10024	0.423	0.67228
Order[High-equal]	-0.19212	0.51807	-0.371	0.71077
Consistency[consistent]:Proportion[High]	0.05830	0.06617	0.881	0.37829
Consistency[consistent]:Order[High-equal]	-0.14301	0.15435	-0.927	0.35418
Proportion[High]:Order[High-equal]	-0.23062	0.08062	-2.860	0.00423
Consistency[consistent]:Proportion[High]:Order[High-equal]	0.07767	0.04512	1.721	0.08516

Threshold coefficients:

	Estimate	Std. Error	z value
1|2	-9.4237	0.5832	-16.160
2|3	-6.3411	0.5360	-11.829
3|4	-4.9764	0.5307	-9.376
4|5	-2.1179	0.5244	-4.039

Table S2: Summary of linear mixed effect model for the N400 (300–600 ms) time window. Summary of model produced by the call `lmer (formula = scale(N400) ~ scale(BL) * Consistency * Proportion * Order * scale(cloze) + (Proportion | subject) + (1 | item), data = cv.N400, REML = FALSE, control = lmerControl(optimizer = "bobyqa", optCtrl = list(maxfun = 1e+06)))`
Linear mixed model fit by maximum likelihood . t-tests use Satterthwaite's method

AIC	BIC	logLik	deviance	df.resid
16275	16528	-8100	16201	7027

Scaled residuals:

Min	1Q	Median	3Q	Max
-5.4	-0.63	-0.01	0.63	4.76

Random effects:

Groups	Name	Variance	Std.Dev.	Corr
item	(Intercept)	0.0032	0.0566	
subject	(Intercept)	0.0398	0.1995	
	Proportion[High]	0.0013	0.0360	-0.11
Residual		0.0567	0.7528	

Number of obs: 7064, groups: item, 160; subject, 48.

Fixed effects:

	Estimate	Std. Error	df	t value	Pr(>|t|)
(Intercept)	−0.0034	0.031	51	−0.11	0.91
scale(BL)	0.62	0.0096	7e+03	64	0
Consistency[consistent]	0.016	0.0095	6.8e+03	1.7	0.086
Proportion[High]	−0.0043	0.012	60	−0.36	0.72
Order[High-equal]	−0.0016	0.03	49	−0.051	0.96
scale(cloze)	−0.0027	0.016	6.3e+02	−0.17	0.87
scale(BL):Consistency[consistent]	0.015	0.0095	7e+03	1.5	0.13
scale(BL):Proportion[High]	−0.0097	0.0096	6.7e+03	−1	0.31
Consistency[consistent]:Proportion[High]	−0.0051	0.0095	6.8e+03	−0.54	0.59
scale(BL):Order[High-equal]	−0.015	0.0096	7e+03	−1.6	0.11
Consistency[consistent]:Order[High-equal]	0.026	0.0095	6.8e+03	2.7	0.0068
Proportion[High]:Order[High-equal]	−0.0026	0.011	55	−0.24	0.81
scale(BL):scale(cloze)	−0.00072	0.015	6.9e+03	−0.048	0.96
Consistency[consistent]:scale(cloze)	0.011	0.016	1.1e+03	0.71	0.48
Proportion[High]:scale(cloze)	0.017	0.016	6.3e+02	1.1	0.27
Order[High-equal]:scale(cloze)	0.0011	0.015	6.8e+03	0.075	0.94
scale(BL):Consistency[consistent]:Proportion[High]	−0.016	0.0095	7e+03	−1.7	0.089
scale(BL):Consistency[consistent]:Order[High-equal]	0.019	0.0095	7e+03	2	0.048
scale(BL):Proportion[High]:Order[High-equal]	−0.0047	0.0095	6.7e+03	−0.5	0.62
Consistency[consistent]:Proportion[High]:Order[High-equal]	0.0014	0.0095	6.8e+03	0.14	0.89
scale(BL):Consistency[consistent]:scale(cloze)	−0.0074	0.015	6.9e+03	−0.49	0.63
scale(BL):Proportion[High]:scale(cloze)	0.02	0.015	6.9e+03	1.3	0.2
Consistency[consistent]:Proportion[High]:scale(cloze)	−0.017	0.016	1.1e+03	−1.1	0.28
scale(BL):Order[High-equal]:scale(cloze)	−0.038	0.015	6.9e+03	−2.5	0.012
Consistency[consistent]:Order[High-equal]:scale(cloze)	−0.016	0.015	6.8e+03	−1.1	0.28
Proportion[High]:Order[High-equal]:scale(cloze)	−0.0012	0.015	6.8e+03	−0.083	0.93
scale(BL):Consistency[consistent]:Proportion[High]:Order[High-equal]	−0.0055	0.0095	7e+03	−0.58	0.56
scale(BL):Consistency[consistent]:Proportion[High]:scale(cloze)	−0.0091	0.015	6.9e+03	−0.6	0.55
scale(BL):Consistency[consistent]:Order[High-equal]:scale(cloze)	0.031	0.015	6.9e+03	2.1	0.04
scale(BL):Proportion[High]:Order[High-equal]:scale(cloze)	0.039	0.015	6.9e+03	2.6	0.0096
Consistency[consistent]:Proportion[High]:Order[High-equal]:scale(cloze)	−0.005	0.015	6.9e+03	−0.33	0.74
scale(BL):Consistency[consistent]:Proportion[High]:Order[High-equal]:scale(cloze)	−0.024	0.015	6.9e+03	−1.6	0.11

Table S3: Analysis of deviance (Type II Wald chi-square tests) for the N400 (300–600 ms) time window.

	Chisq	Df	Pr(>Chisq)
scale(BL)	4591.712	1	0.000
Consistency	2.249	1	0.134
Proportion	0.553	1	0.457
Order	0.021	1	0.884
scale(cloze)	0.670	1	0.413
scale(BL):Consistency	1.999	1	0.157
scale(BL):Proportion	1.647	1	0.199
Consistency:Proportion	0.054	1	0.816
scale(BL):Order	0.681	1	0.409
Consistency:Order	8.285	1	0.004
Proportion:Order	0.120	1	0.729
scale(BL):scale(cloze)	0.787	1	0.375
Consistency:scale(cloze)	0.004	1	0.947
Proportion:scale(cloze)	0.212	1	0.645
Order:scale(cloze)	0.844	1	0.358
scale(BL):Consistency:Proportion	2.496	1	0.114
scale(BL):Consistency:Order	2.305	1	0.129
scale(BL):Proportion:Order	1.345	1	0.246
Consistency:Proportion:Order	0.069	1	0.793
scale(BL):Consistency:scale(cloze)	2.054	1	0.152
scale(BL):Proportion:scale(cloze)	1.105	1	0.293
Consistency:Proportion:scale(cloze)	0.820	1	0.365
scale(BL):Order:scale(cloze)	0.321	1	0.571
Consistency:Order:scale(cloze)	3.919	1	0.048
Proportion:Order:scale(cloze)	0.224	1	0.636
scale(BL):Consistency:Proportion:Order	0.038	1	0.845
scale(BL):Consistency:Proportion:scale(cloze)	0.208	1	0.648
scale(BL):Consistency:Order:scale(cloze)	1.667	1	0.197
scale(BL):Proportion:Order:scale(cloze)	4.170	1	0.041
Consistency:Proportion:Order:scale(cloze)	0.059	1	0.808
scale(BL):Consistency:Proportion:Order:scale(cloze)	2.558	1	0.110

Table S4: Summary of linear mixed effect model with trials as an additional predictor for the N400 (300–600ms) time window. Summary of model produced by the call `lmer(formula = scale(N400) ~ scale(BL) * Consistency * Proportion * Order * (scale(cloze) + scale(Trials)) + (Proportion | subject) + (1 | item), data = cv.N400, REML = FALSE, control = lmerControl(optimizer = "bobyqa", optCtrl = list(maxfun = 1e+06)))`
Linear mixed model fit by maximum likelihood . t-tests use Satterthwaite's method

AIC	BIC	logLik	deviance	df.resid
16271	16635	-8083	16165	7011

Scaled residuals:

Min	1Q	Median	3Q	Max
-5.35	-0.62	-0.01	0.63	4.77

Random effects:

Groups	Name	Variance	Std.Dev.	Corr
item	(Intercept)	0.0026	0.0514	
subject	(Intercept)	0.0395	0.1988	
	Proportion[High]	0.0014	0.0370	-0.10
Residual		0.5642	0.7512	

Number of obs: 7064, groups: item, 160; subject, 48.

Fixed effects:

	Estimate	Std. Error	df	t value	Pr(>|t|)
(Intercept)	−0.0098	0.034	80	−0.29	0.78
scale(BL)	0.63	0.019	7e+03	34	1.1e−230
Consistency[consistent]	−0.015	0.018	7e+03	−0.82	0.41
Proportion[High]	−0.019	0.019	4.4e+02	−0.96	0.34
Order[High-equal]	−0.01	0.035	82	−0.29	0.77
scale(cloze)	−0.0023	0.016	6.1e+02	−0.14	0.89
scale(Trials)	−0.03	0.02	1.6e+02	−1.5	0.13
scale(BL):Consistency[consistent]	−0.045	0.019	7e+03	−2.4	0.016
scale(BL):Proportion[High]	−0.017	0.019	7e+03	−0.92	0.36
Consistency[consistent]:Proportion[High]	0.0071	0.018	7e+03	0.39	0.7
scale(BL):Order[High-equal]	−0.008	0.019	7e+03	−0.42	0.67
Consistency[consistent]:Order[High-equal]	0.043	0.018	6.9e+03	2.4	0.018
Proportion[High]:Order[High-equal]	−0.029	0.02	2.1e+02	−1.4	0.16
scale(BL):scale(cloze)	−0.0043	0.015	6.9e+03	−0.28	0.78
scale(BL):scale(Trials)	0.046	0.019	7e+03	2.5	0.014
Consistency[consistent]:scale(cloze)	0.015	0.016	1e+03	0.96	0.34
Consistency[consistent]:scale(Trials)	−0.031	0.018	6.9e+03	−1.7	0.084
Proportion[High]:scale(cloze)	0.019	0.016	6.1e+02	1.2	0.23
Proportion[High]:scale(Trials)	−0.01	0.02	1.6e+02	−0.51	0.61
Order[High-equal]:scale(cloze)	0.0044	0.015	6.8e+03	0.29	0.77
Order[High-equal]:scale(Trials)	−0.016	0.018	6.9e+03	−0.9	0.37
scale(BL):Consistency[consistent]:Proportion[High]	0.0063	0.019	7e+03	0.34	0.74
scale(BL):Consistency[consistent]:Order[High-equal]	0.0099	0.019	7e+03	0.53	0.6
scale(BL):Proportion[High]:Order[High-equal]	0.035	0.019	7e+03	1.8	0.064
Consistency[consistent]:Proportion[High]:Order[High-equal]	−0.025	0.018	6.9e+03	−1.4	0.17
scale(BL):Consistency[consistent]:scale(cloze)	−0.0055	0.015	7e+03	−0.36	0.72
scale(BL):Consistency[consistent]:scale(Trials)	−0.0036	0.019	7e+03	−0.2	0.84
scale(BL):Proportion[High]:scale(cloze)	0.022	0.015	6.9e+03	1.4	0.15
scale(BL):Proportion[High]:scale(Trials)	0.0086	0.019	7e+03	0.46	0.64
Consistency[consistent]:Proportion[High]:scale(cloze)	−0.019	0.016	1e+03	−1.2	0.23
Consistency[consistent]:Proportion[High]:scale(Trials)	0.021	0.018	7e+03	1.2	0.25
scale(BL):Order[High-equal]:scale(cloze)	−0.031	0.015	6.9e+03	−2	0.042
scale(BL):Order[High-equal]:scale(Trials)	−0.0096	0.019	7e+03	−0.52	0.61
Consistency[consistent]:Order[High-equal]:scale(cloze)	−0.02	0.015	6.8e+03	−1.3	0.19
Consistency[consistent]:Order[High-equal]:scale(Trials)	0.015	0.018	6.8e+03	0.81	0.42
Proportion[High]:Order[High-equal]:scale(cloze)	−0.0025	0.015	6.8e+03	−0.17	0.87
Proportion[High]:Order[High-equal]:scale(Trials)	−0.007	0.018	6.9e+03	−0.38	0.7
scale(BL):Consistency[consistent]:Proportion[High]:Order[High-equal]	−0.0079	0.019	7e+03	−0.42	0.67
scale(BL):Consistency[consistent]:Proportion[High]:scale(cloze)	−0.013	0.015	7e+03	−0.87	0.38
scale(BL):Consistency[consistent]:Proportion[High]:scale(Trials)	−0.0099	0.019	7e+03	−0.53	0.6
scale(BL):Consistency[consistent]:Order[High-equal]:scale(cloze)	0.023	0.015	6.9e+03	1.5	0.13
scale(BL):Consistency[consistent]:Order[High-equal]:scale(Trials)	0.025	0.019	7e+03	1.3	0.19
scale(BL):Proportion[High]:Order[High-equal]:scale(cloze)	0.034	0.015	6.9e+03	2.2	0.028
scale(BL):Proportion[High]:Order[High-equal]:scale(Trials)	0.016	0.019	7e+03	0.86	0.39
Consistency[consistent]:Proportion[High]:Order[High-equal]:scale(cloze)	1.1e−05	0.015	6.9e+03	0.00073	1
Consistency[consistent]:Proportion[High]:Order[High-equal]:scale(Trials)	−0.037	0.018	6.8e+03	−2	0.042
scale(BL):Consistency[consistent]:Proportion[High]:Order[High-equal]:scale(cloze)	−0.015	0.015	6.9e+03	−0.97	0.33
scale(BL):Consistency[consistent]:Proportion[High]:Order[High-equal]:scale(Trials)	−0.069	0.019	7e+03	−3.7	0.0002

Table S5: Analysis of deviance (Type II Wald chi-square tests) in the N400 (300–600 ms) time window for models with trials as an additional predictor.

	Chisq	Df	Pr(>Chisq)
scale(BL)	4616.930	1	0.000
Consistency	2.170	1	0.141
Proportion	1.312	1	0.252
Order	0.178	1	0.673
scale(cloze)	1.207	1	0.272
scale(Trials)	2.230	1	0.135
scale(BL):Consistency	1.944	1	0.163
scale(BL):Proportion	1.502	1	0.220
Consistency:Proportion	0.516	1	0.472
scale(BL):Order	0.008	1	0.929
Consistency:Order	5.682	1	0.017
Proportion:Order	2.081	1	0.149
scale(BL):scale(cloze)	0.489	1	0.484
scale(BL):scale(Trials)	6.313	1	0.012
Consistency:scale(cloze)	0.110	1	0.741
Consistency:scale(Trials)	2.629	1	0.105
Proportion:scale(cloze)	0.241	1	0.623
Proportion:scale(Trials)	0.299	1	0.585
Order:scale(cloze)	0.515	1	0.473
Order:scale(Trials)	0.810	1	0.368
scale(BL):Consistency:Proportion	0.139	1	0.709
scale(BL):Consistency:Order	0.319	1	0.572
scale(BL):Proportion:Order	2.602	1	0.107
Consistency:Proportion:Order	1.683	1	0.194
scale(BL):Consistency:scale(cloze)	2.266	1	0.132
scale(BL):Consistency:scale(Trials)	0.175	1	0.676
scale(BL):Proportion:scale(cloze)	1.067	1	0.302
scale(BL):Proportion:scale(Trials)	0.114	1	0.736
Consistency:Proportion:scale(cloze)	1.157	1	0.282
Consistency:Proportion:scale(Trials)	1.254	1	0.263
scale(BL):Order:scale(cloze)	0.169	1	0.681
scale(BL):Order:scale(Trials)	0.228	1	0.633
Consistency:Order:scale(cloze)	3.939	1	0.047
Consistency:Order:scale(Trials)	0.959	1	0.327
Proportion:Order:scale(cloze)	0.061	1	0.804
Proportion:Order:scale(Trials)	0.164	1	0.686
scale(BL):Consistency:Proportion:Order	0.159	1	0.690
scale(BL):Consistency:Proportion:scale(cloze)	0.632	1	0.427
scale(BL):Consistency:Proportion:scale(Trials)	0.131	1	0.717
scale(BL):Consistency:Order:scale(cloze)	1.407	1	0.235
scale(BL):Consistency:Order:scale(Trials)	1.356	1	0.244
scale(BL):Proportion:Order:scale(cloze)	4.270	1	0.039
scale(BL):Proportion:Order:scale(Trials)	1.019	1	0.313
Consistency:Proportion:Order:scale(cloze)	0.004	1	0.953
Consistency:Proportion:Order:scale(Trials)	4.268	1	0.039
scale(BL):Consistency:Proportion:Order:scale(cloze)	0.935	1	0.334
scale(BL):Consistency:Proportion:Order:scale(Trials)	13.853	1	0.000

Table S6: Summary of linear mixed effect model for the LN (650–1000 ms) time window. Summary of model produced by the call `lmer(formula = scale(LN) ~ scale(BL) * Consistency * Proportion * Order * scale(cloze) + (Proportion | subject) + (1 | item), data = cv.LN, REML = FALSE, control = lmerControl(optimizer = "bobyqa", optCtrl = list(maxfun = 1e+06)))`
Linear mixed model fit by maximum likelihood . t-tests use Satterthwaite's method

AIC	BIC	logLik	deviance	df.resid
18055	18309	-8990	17981	7027

Scaled residuals:

Min	1Q	Median	3Q	Max
-5.02	-0.61	-0.01	0.62	6.07

Random effects:

Groups	Name	Variance	Std.Dev.	Corr
item	(Intercept)	0.0000	0.0000	
subject	(Intercept)	0.02888	0.1699	
	Proportion[High]	0.0008	0.0282	-0.38
Residual		0.7361	0.8580	

Number of obs: 7064, groups: item, 160; subject, 48.

Fixed effects:

	Estimate	Std. Error	df	t value	Pr(>|t|)
(Intercept)	−0.0054	0.027	49	−0.2	0.84
scale(BL)	0.46	0.011	7.1e+03	42	0
Consistency[consistent]	0.018	0.011	7e+03	1.7	0.094
Proportion[High]	0.014	0.012	57	1.2	0.22
Order[High-equal]	0.034	0.027	49	1.3	0.21
scale(cloze)	−0.041	0.017	7e+03	−2.4	0.018
scale(BL):Consistency[consistent]	0.02	0.011	7e+03	1.9	0.064
scale(BL):Proportion[High]	−0.0097	0.011	6.7e+03	−0.9	0.37
Consistency[consistent]:Proportion[High]	−0.00053	0.011	7e+03	−0.049	0.96
scale(BL):Order[High-equal]	−0.028	0.011	7.1e+03	−2.6	0.01
Consistency[consistent]:Order[High-equal]	0.025	0.011	7e+03	2.4	0.018
Proportion[High]:Order[High-equal]	−0.02	0.012	57	−1.7	0.093
scale(BL):scale(cloze)	−0.013	0.017	7e+03	−0.74	0.46
Consistency[consistent]:scale(cloze)	0.024	0.017	7e+03	1.4	0.17
Proportion[High]:scale(cloze)	0.039	0.017	7e+03	2.3	0.022
Order[High-equal]:scale(cloze)	−0.00073	0.017	7e+03	−0.042	0.97
scale(BL):Consistency[consistent]:Proportion[High]	−0.0084	0.011	7e+03	−0.78	0.44
scale(BL):Consistency[consistent]:Order[High-equal]	0.031	0.011	7e+03	2.9	0.004
scale(BL):Proportion[High]:Order[High-equal]	0.02	0.011	6.7e+03	1.8	0.07
Consistency[consistent]:Proportion[High]:Order[High-equal]	−0.0051	0.011	7e+03	−0.48	0.63
scale(BL):Consistency[consistent]:scale(cloze)	0.034	0.017	7e+03	2	0.049
scale(BL):Proportion[High]:scale(cloze)	0.044	0.017	7e+03	2.6	0.011
Consistency[consistent]:Proportion[High]:scale(cloze)	−0.028	0.017	7e+03	−1.6	0.11
scale(BL):Order[High-equal]:scale(cloze)	−0.088	0.017	7e+03	−5.2	2.4e−07
Consistency[consistent]:Order[High-equal]:scale(cloze)	−0.016	0.017	7e+03	−0.95	0.34
Proportion[High]:Order[High-equal]:scale(cloze)	−0.01	0.017	7e+03	−0.61	0.54
scale(BL):Consistency[consistent]:Proportion[High]:Order[High-equal]	−0.0014	0.011	7e+03	−0.13	0.89
scale(BL):Consistency[consistent]:Proportion[High]:scale(cloze)	−0.042	0.017	7e+03	−2.5	0.013
scale(BL):Consistency[consistent]:Order[High-equal]:scale(cloze)	0.088	0.017	7e+03	5.2	2.6e−07
scale(BL):Proportion[High]:Order[High-equal]:scale(cloze)	0.096	0.017	7e+03	5.6	2.2e−08
Consistency[consistent]:Proportion[High]:Order[High-equal]:scale(cloze)	0.0064	0.017	7e+03	0.37	0.71
scale(BL):Consistency[consistent]:Proportion[High]:Order[High-equal]:scale(cloze)	−0.079	0.017	7e+03	−4.6	3.6e−06

Table S7: Analysis of deviance (Type II Wald chi-square tests) for the LN (650–1000 ms) time window.

	Chisq	Df	Pr(>Chisq)
scale(BL)	2000.519	1	0.000
Consistency	1.339	1	0.247
Proportion	0.405	1	0.525
Order	0.927	1	0.336
scale(cloze)	1.854	1	0.173
scale(BL):Consistency	1.438	1	0.231
scale(BL):Proportion	3.210	1	0.073
Consistency:Proportion	0.266	1	0.606
scale(BL):Order	0.618	1	0.432
Consistency:Order	6.177	1	0.013
Proportion:Order	2.920	1	0.087
scale(BL):scale(cloze)	5.694	1	0.017
Consistency:scale(cloze)	0.041	1	0.840
Proportion:scale(cloze)	1.910	1	0.167
Order:scale(cloze)	1.632	1	0.201
scale(BL):Consistency:Proportion	0.037	1	0.847
scale(BL):Consistency:Order	2.538	1	0.111
scale(BL):Proportion:Order	0.079	1	0.779
Consistency:Proportion:Order	0.228	1	0.633
scale(BL):Consistency:scale(cloze)	0.001	1	0.982
scale(BL):Proportion:scale(cloze)	0.966	1	0.326
Consistency:Proportion:scale(cloze)	1.185	1	0.276
scale(BL):Order:scale(cloze)	0.315	1	0.575
Consistency:Order:scale(cloze)	1.426	1	0.232
Proportion:Order:scale(cloze)	0.447	1	0.504
scale(BL):Consistency:Proportion:Order	1.005	1	0.316
scale(BL):Consistency:Proportion:scale(cloze)	4.117	1	0.042
scale(BL):Consistency:Order:scale(cloze)	6.564	1	0.010
scale(BL):Proportion:Order:scale(cloze)	11.784	1	0.001
Consistency:Proportion:Order:scale(cloze)	0.514	1	0.473
scale(BL):Consistency:Proportion:Order:scale(cloze)	21.496	1	0.000

Table S8: Summary of linear mixed effect model with trials as an additional predictor for the LN (650–1000 ms) time window. Summary of model produced by the call `lmer(formula = scale(LN) ~ scale(BL) * Consistency * Proportion * Order * (scale(cloze) + scale(Trials)) + (Proportion | subject) + (1 | item), data = cv.LN, REML = FALSE, control = lmerControl(optimizer = "bobyqa", optCtrl = list(maxfun = 1e+06)))`
Linear mixed model fit by maximum likelihood . t-tests use Satterthwaite's method

AIC	BIC	logLik	deviance	df.resid
18061	18424	-8977	17955	7011

Scaled residuals:

Min	1Q	Median	3Q	Max
-5.04	-0.6	-0.02	0.62	6.1

Random effects:

Groups	Name	Variance	Std.Dev.	Corr
item	(Intercept)	1.108e-15	3.33e-08	
subject	(Intercept)	2.908e-02	1.71e-01	
	Proportion[High]	9.658e-04	3.11e-02	-0.32
Residual		7.332e-01	8.56e-01	

Number of obs: 7064, groups: item, 160; subject, 48.

Fixed effects:

	Estimate	Std. Error	df	t value	Pr(>|t|)
(Intercept)	−0.014	0.032	1e+02	−0.42	0.67
scale(BL)	0.48	0.021	7e+03	23	1.8e−108
Consistency[consistent]	−0.00036	0.021	7e+03	−0.017	0.99
Proportion[High]	0.022	0.021	5.9e+02	1	0.3
Order[High-equal]	0.02	0.032	1e+02	0.63	0.53
scale(cloze)	−0.038	0.017	7e+03	−2.2	0.027
scale(Trials)	−0.037	0.021	7e+03	−1.8	0.072
scale(BL):Consistency[consistent]	−0.019	0.021	7e+03	−0.91	0.36
scale(BL):Proportion[High]	−0.01	0.021	7e+03	−0.48	0.63
Consistency[consistent]:Proportion[High]	0.0065	0.021	7e+03	0.32	0.75
scale(BL):Order[High-equal]	−0.024	0.021	7e+03	−1.1	0.25
Consistency[consistent]:Order[High-equal]	0.022	0.021	7e+03	1.1	0.28
Proportion[High]:Order[High-equal]	−0.052	0.021	5.9e+02	−2.4	0.015
scale(BL):scale(cloze)	−0.012	0.017	7e+03	−0.72	0.47
scale(BL):scale(Trials)	0.027	0.021	7e+03	1.3	0.2
Consistency[consistent]:scale(cloze)	0.029	0.017	7e+03	1.7	0.094
Consistency[consistent]:scale(Trials)	−0.066	0.021	7e+03	−3.2	0.0014
Proportion[High]:scale(cloze)	0.042	0.017	7e+03	2.5	0.014
Proportion[High]:scale(Trials)	−0.016	0.021	7e+03	−0.75	0.45
Order[High-equal]:scale(cloze)	−1.3e−05	0.017	7e+03	−0.00074	1
Order[High-equal]:scale(Trials)	0.0094	0.021	7e+03	0.45	0.65
scale(BL):Consistency[consistent]:Proportion[High]	−0.012	0.021	7e+03	−0.56	0.57
scale(BL):Consistency[consistent]:Order[High-equal]	0.00094	0.021	7e+03	0.044	0.96
scale(BL):Proportion[High]:Order[High-equal]	0.042	0.021	7e+03	2	0.045
Consistency[consistent]:Proportion[High]:Order[High-equal]	−0.061	0.021	7e+03	−2.9	0.0032
scale(BL):Consistency[consistent]:scale(cloze)	0.034	0.017	7e+03	2	0.049
scale(BL):Consistency[consistent]:scale(Trials)	−0.0058	0.021	7e+03	−0.27	0.78
scale(BL):Proportion[High]:scale(cloze)	0.044	0.017	7e+03	2.6	0.01
scale(BL):Proportion[High]:scale(Trials)	0.0043	0.021	7e+03	0.21	0.84
Consistency[consistent]:Proportion[High]:scale(cloze)	−0.028	0.017	7e+03	−1.6	0.1
Consistency[consistent]:Proportion[High]:scale(Trials)	−0.004	0.021	7e+03	−0.2	0.85
scale(BL):Order[High-equal]:scale(cloze)	−0.084	0.017	7e+03	−4.9	1.3e−06
scale(BL):Order[High-equal]:scale(Trials)	−0.00038	0.021	7e+03	−0.018	0.99
Consistency[consistent]:Order[High-equal]:scale(cloze)	−0.018	0.017	7e+03	−1	0.29
Consistency[consistent]:Order[High-equal]:scale(Trials)	0.0088	0.021	7e+03	0.42	0.67
Proportion[High]:Order[High-equal]:scale(cloze)	−0.01	0.017	7e+03	−0.59	0.56
Proportion[High]:Order[High-equal]:scale(Trials)	−0.009	0.021	7e+03	−0.43	0.67
scale(BL):Consistency[consistent]:Proportion[High]:Order[High-equal]	−0.0059	0.021	7e+03	−0.28	0.78
scale(BL):Consistency[consistent]:Proportion[High]:scale(cloze)	−0.043	0.017	7e+03	−2.5	0.013
scale(BL):Consistency[consistent]:Proportion[High]:scale(Trials)	−0.035	0.021	7e+03	−1.7	0.095
scale(BL):Consistency[consistent]:Order[High-equal]:scale(cloze)	0.084	0.017	7e+03	4.8	1.4e−06
scale(BL):Consistency[consistent]:Order[High-equal]:scale(Trials)	−0.0055	0.021	7e+03	−0.26	0.79
scale(BL):Proportion[High]:Order[High-equal]:scale(cloze)	0.093	0.017	7e+03	5.4	7.2e−08
scale(BL):Proportion[High]:Order[High-equal]:scale(Trials)	0.027	0.021	7e+03	1.3	0.21
Consistency[consistent]:Proportion[High]:Order[High-equal]:scale(cloze)	0.0084	0.017	7e+03	0.49	0.63
Consistency[consistent]:Proportion[High]:Order[High-equal]:scale(Trials)	−0.022	0.021	7e+03	−1.1	0.28
scale(BL):Consistency[consistent]:Proportion[High]:Order[High-equal]:scale(cloze)	−0.074	0.017	7e+03	−4.3	1.8e−05
scale(BL):Consistency[consistent]:Proportion[High]:Order[High-equal]:scale(Trials)	−0.046	0.021	7e+03	−2.2	0.03

Table S9: Analysis of deviance (Type II Wald chi-square tests) in the LN (600–1000 ms) time window for models with trials as an additional predictor.

	Chisq	Df	Pr(>Chisq)
scale(BL)	2010.553	1	0.000
Consistency	1.274	1	0.259
Proportion	0.558	1	0.455
Order	0.171	1	0.679
scale(cloze)	0.590	1	0.442
scale(Trials)	3.089	1	0.079
scale(BL):Consistency	1.551	1	0.213
scale(BL):Proportion	1.657	1	0.198
Consistency:Proportion	0.621	1	0.431
scale(BL):Order	0.078	1	0.780
Consistency:Order	1.192	1	0.275
Proportion:Order	5.676	1	0.017
scale(BL):scale(cloze)	5.790	1	0.016
scale(BL):scale(Trials)	1.830	1	0.176
Consistency:scale(cloze)	0.355	1	0.551
Consistency:scale(Trials)	9.573	1	0.002
Proportion:scale(cloze)	2.520	1	0.112
Proportion:scale(Trials)	0.599	1	0.439
Order:scale(cloze)	1.506	1	0.220
Order:scale(Trials)	0.175	1	0.675
scale(BL):Consistency:Proportion	0.024	1	0.878
scale(BL):Consistency:Order	0.224	1	0.636
scale(BL):Proportion:Order	1.687	1	0.194
Consistency:Proportion:Order	8.717	1	0.003
scale(BL):Consistency:scale(cloze)	0.002	1	0.967
scale(BL):Consistency:scale(Trials)	0.185	1	0.667
scale(BL):Proportion:scale(cloze)	1.037	1	0.309
scale(BL):Proportion:scale(Trials)	0.037	1	0.848
Consistency:Proportion:scale(cloze)	1.334	1	0.248
Consistency:Proportion:scale(Trials)	0.048	1	0.827
scale(BL):Order:scale(cloze)	0.186	1	0.666
scale(BL):Order:scale(Trials)	0.007	1	0.935
Consistency:Order:scale(cloze)	1.506	1	0.220
Consistency:Order:scale(Trials)	0.271	1	0.603
Proportion:Order:scale(cloze)	0.283	1	0.595
Proportion:Order:scale(Trials)	0.199	1	0.656
scale(BL):Consistency:Proportion:Order	0.022	1	0.881
scale(BL):Consistency:Proportion:scale(cloze)	4.332	1	0.037
scale(BL):Consistency:Proportion:scale(Trials)	2.440	1	0.118
scale(BL):Consistency:Order:scale(cloze)	6.109	1	0.013
scale(BL):Consistency:Order:scale(Trials)	0.124	1	0.725
scale(BL):Proportion:Order:scale(cloze)	11.886	1	0.001
scale(BL):Proportion:Order:scale(Trials)	1.900	1	0.168
Consistency:Proportion:Order:scale(cloze)	0.666	1	0.414
Consistency:Proportion:Order:scale(Trials)	1.220	1	0.269
scale(BL):Consistency:Proportion:Order:scale(cloze)	18.365	1	0.000
scale(BL):Consistency:Proportion:Order:scale(Trials)	4.730	1	0.030
